# Low CO_2_ induces urea cycle intermediate accumulation in *Arabidopsis thaliana*

**DOI:** 10.1371/journal.pone.0210342

**Published:** 2019-01-16

**Authors:** Christian Blume, Julia Ost, Marco Mühlenbruch, Christoph Peterhänsel, Miriam Laxa

**Affiliations:** Institute of Botany, Leibniz University Hannover, Hannover, Germany; Universidade Federal de Vicosa, BRAZIL

## Abstract

The non-proteinogenic amino acid ornithine links several stress response pathways. From a previous study we know that ornithine accumulates in response to low CO_2_. To investigate ornithine accumulation in plants, we shifted plants to either low CO_2_ or low light. Both conditions increased carbon limitation, but only low CO_2_ also increased the rate of photorespiration. Changes in metabolite profiles of light- and CO_2_-limited plants were quite similar. Several amino acids that are known markers of senescence accumulated strongly under both conditions. However, urea cycle intermediates respond differently between the two treatments. While the levels of both ornithine and citrulline were much higher in plants shifted to 100 ppm CO_2_ compared to those kept in 400 ppm CO_2_, their metabolite abundance did not significantly change in response to a light limitation. Furthermore, both ornithine and citrulline accumulation is independent from sugar starvation. Exogenous supplied sugar did not significantly change the accumulation of the two metabolites in low CO_2_-stressed plants, while the accumulation of other amino acids was reduced by about 50%. Gene expression measurements showed a reduction of the entire arginine biosynthetic pathway in response to low CO_2_. Genes in both proline biosynthesis and degradation were induced. Hence, proline did not accumulate in response to low CO_2_ like observed for many other stresses. We propose that excess of nitrogen re-fixed during photorespiration can be alternatively stored in ornithine and citrulline under low CO_2_ conditions. Furthermore, ornithine is converted to pyrroline-5-carboxylate by the action of δOAT.

## Introduction

Photosynthesis drives the fixation of carbon dioxide in the Calvin-Benson cycle. This process enables plants to build up sugars. During their life cycle, plants are subjected to several kinds of stress that can limit the net carboxylation rate of ribulose-1,5-bisphosphate carboxylase/oxygenase (RuBisCO). For example, dependent on light intensity the carboxylation rate shows a linear behavior for low light intensities, while the catalytic activity of RuBisCO is the rate-limiting factor at higher light intensities [1]. Furthermore, the rate of carboxylation is a function of substrate availability. For instance, drought or heat stress limit the CO_2_ availability due to stomatal closure in order to prevent an increase in transpiration [2]. Consequently, the amount of CO_2_ that can be fixed by RuBisCO is the limiting factor. In addition, CO_2_ limiting conditions lead to an increase in oxygen fixation by RuBisCO. Therefore, flux through the photorespiratory cycle is increased. Photorespiration goes along with the production of reactive oxygen species especially in peroxisomes by glycolate oxidase (GOX) [3]. Furthermore, the mitochondrial glycine decarboxylase complex produces ammonia during photorespiration that is re-fixed by the sequential action of glutamate synthase (GLU1) and glutamine synthetase (GS2) [3]. CO_2_ limiting conditions can be mimicked by low CO_2_ treatments in the lab. Low CO_2_ induces a variety of phenotypical changes in *Arabidopsis*. Short term limited CO_2_ responses, here represented by an 8 h shift from 1% CO_2_ to 0.038% CO_2_, did not lead to changes in the visible phenotype, but induces molecular changes [4]. While only five genes significantly responded (*q* < 0.01) to the CO_2_ shift, metabolites related to photorespiration (glycerate, glycolate, serine and glycine) were significantly induced [4]. Furthermore, both the transpiration rate and ABA levels were increased in plants facing limiting CO_2_ conditions [4]. Long term low CO_2_ responses (6-weeks grown under 100 ppm compared to 380 ppm control conditions) led to a significant visible phenotype that appeared as extreme impact on plant growth [5]. In addition, plants showed a delayed flowering, a decrease in productivity, and an increase in stomatal density. On molecular level, the size and arrangement of bundle sheath and mesophyll cells was unchanged, but low CO_2_ led to a decrease in grana stacking in both cell types [5]. Transcript analysis revealed an upregulation of photorespiratory genes which did not exceed 2-fold [5]. In a previous publication, we observed that ornithine accumulated when plants were shifted from ambient CO_2_ (400 ppm) to low CO_2_ (100 ppm CO_2_) concentrations [6]. However, both the source and the reason for the accumulation of ornithine have not been investigated to date.

**Fig 1:**
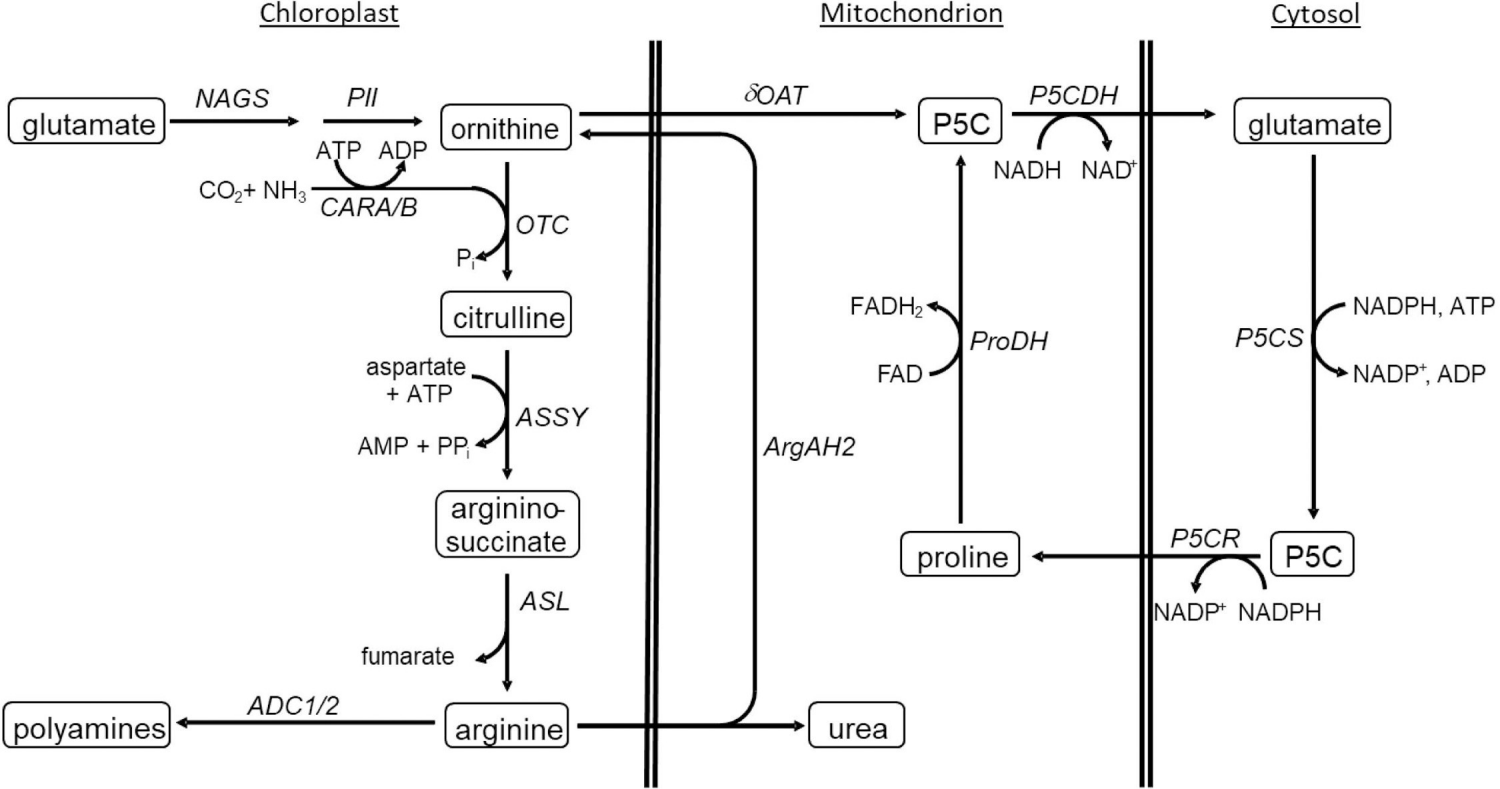
Overview on ornithine synthesis and degradation. ADC1/2, arginine decarboxylase 1 and 2; ArgAH, arginine amidohydrolase; ASL, argininosuccinate lyase; ASSY, argininosuccinate synthetase; CARA, carbamoyl phosphate synthetase A; CARB, carbamoyl phosphate synthetase B; NAGS, N-acetyl-L-glutamate synthase; δOAT, ornithine-δ-aminotransferase; OTC, ornithine transcarbamylase; PII, PII protein; P5CDH, pyrroline-5-carboxylate dehydrogenase; P5CS, pyrroline-5-carboxylate synthetase; P5CR, pyrroline-5-carboxylate reductase; PRODH, proline dehydrogenase.

Ornithine is an intermediate of the urea cycle and, thus, a central metabolite of arginine synthesis and degradation [7] (Fig 1). Arginine synthesis from ornithine takes place in chloroplasts [8]. In a first step, ornithine is carbamoylated to citrulline. Carbamoyl-P is synthesized from either glutamine or ammonia and bicarbonate in the presence of ATP by the heteromeric enzyme carbamoyl phosphate synthetase (CPS) that is encoded by the two genes *CARA* and *CARB* in *Arabidopsis* [8]. Subsequently, argininosuccinate synthetase (ASS) catalyzes the energy-dependent synthesis of argininosuccinate from citrulline and aspartate. Release of fumarate by the action of argininosuccinate lyase (ASL) converts argininosuccinate into arginine. In turn, breakdown of arginine by arginase releases urea and produces ornithine. This reaction is catalyzed by two mitochondrial arginases in *Arabidopsis* [9]. Urea can be further degraded to CO_2_ and NH_3_ by cytosolic urease.

Ornithine can also be synthesized from glutamate in five reaction steps [8] (Fig 1). There is evidence that all reaction steps are localized in the chloroplast stroma. Although the rate limiting step of this pathway is catalyzed by N-acetyl-L-glutamate synthase (NAGS) [10], regulation of the pathway takes place at the next enzymatic step in the cascade that is catalyzed by N-acetyl-L-glutamate kinase (NAGK). NAGK is a target of feedback inhibition by arginine, whereas N-acetyl glutamate restores its activity [11,12]. Binding of arginine to NAGK can be reversed by complex formation with the PII protein that was identified as major regulator of ornithine synthesis [13]. Complex formation of PII and NAGK is dependent on the binding of glutamine and α-ketoglutarate that enable and disable binding of PII to NAGK, respectively [12,14]. In bacteria, PII is the major regulator for balancing the C/N ratio. Its importance in plants is still discussed, but the interaction with two of the major actors in the production of organic nitrogen, a-ketoglutarate and glutamine, implicates a similar potential as in bacteria [15,16].

In addition to arginine production, ornithine is a precursor of the polyamines putrescine, spermine and spermidine (Fig 1). Decarboxylation of either ornithine or arginine initializes their production. Because ornithine decarboxylase seems to be absent from the *Arabidopsis thaliana* genome, arginine decarboxylase remains to be the sole source for polyamine synthesis [17].

Ornithine is also a potential precursor of proline synthesis (Fig 1). Ornithine-δ-aminotransferase (δOAT) converts ornithine to glutamate semialdehyde which reacts non-enzymatically to pyrroline-5-carboxylate (P5C). P5C can be further converted to glutamate by pyrroline-5-carboxylate dehydrogenase (P5CDH), transported to the cytosol where it can be converted to proline by pyrroline-5-carboxylate synthase (P5CS). It is still discussed, whether this reaction is used to build up proline under stress conditions or whether this enzymes just catalyzes an intermediate step in arginine degradation to form glutamate [7].

Niessen and colleagues [6] observed ornithine accumulation after shifting *Arabidopsis thaliana* plants from 400 ppm to 100 ppm CO_2_. A low CO_2_ treatment imposes several stresses like carbohydrate starvation, a high photorespiration rate, and the generation of ROS [4,18,19]. The aim of this study was to analyze the accumulation of ornithine by correlating metabolite changes to changes in gene expression in ornithine biosynthetic and degradation pathways.

## Methods

### Plant growth

*Arabidopsis thaliana* Col-0 plants were grown in GroBanks (CLF Plant Climatics, Wertingen) with a light intensity of 150 μmol m^−2^ s^−1^ (μE) under short-day conditions (8 h illumination, 22°C, and 16 h darkness, 20°C). After 5 weeks, half of the plants were shifted to either 100 ppm CO_2_ or a light intensity of 20 μmol m^−2^ s^−1^ before the onset of light. The control group was kept at ambient 400 ppm CO_2_ and a light intensity of 150 μmol m^−2^ s^−1^, respectively. Samples (three rosettes per treatment) were harvested and snap frozen in liquid nitrogen at the time points indicated in the figure legends.

For the sucrose treatment, plants were grown on ½ MS (Murashige and Skoog, Duchefa, Haarlem, Netherlands) supplemented with vitamins and 2% sucrose. MS medium of the control group was only supplemented with vitamins. After three weeks, half of the plants of the individual treatments was shifted to 100 ppm CO_2_ before the onset of light. The four groups of differently treated plants were as followed: i) no sucrose, 400 ppm CO_2_, ii) no sucrose, 100 ppm CO_2_, iii) 2% sucrose, 400 ppm CO_2_ and iv) 2% sucrose, 100 ppm CO_2_. Samples (six plantlets per treatment) were harvested and snap frozen in liquid nitrogen at the time points indicated in the figure legends.

### RNA isolation and quantitative RT-PCR

RNA was isolated following a modified protocol from Weckwerth and colleagues [20]. About 20 mg ground leaf material was dissolved in 500 μl extraction buffer [50 mM Tris-HCl, pH 7.5, 0.5% w/v SDS]. 500 μl water saturated phenol were added followed by 10 min shaking at room temperature. After phase separation (16,000*g* at RT for 10 min) 300 μl of the upper phase were transferred into a new tube, and mixed with two volumes 96% EtOH and 1/10 volume sodium acetate (3 M, pH 5.2). DNA and RNA were precipitated by centrifugation at 16,000 g and 4°C for 20 min. The pellet was washed with 70% EtOH und purity was determined by measuring the ratio of 260 nm and 280 nm. cDNA synthesis and quantitative PCR were done as described by Niessen and colleagues [6]. Gene specific primers are listed in Table 1.

**Table 1:** Primer used for measurement of gene transcription.

Gene	AGI-code	Forward primer	Reverse primer
*ADC1*	AT2G16500	CATGGAAACGTCAACAAACGCTC	CCAATTCTCATCTTTGCCCTTGC
*ADC2*	AT4G34710	CAACAATGTGGCGGCTTCTCTC	CGATGCCTGCTCAGTTGCAAG
*ArgAH1*	AT4G08900	CGGCATTTGCACCTGGAGTGT	TGCGGGTTGAACTCGACAACG
*ArgAH2*	AT4G08870	CCTTGCGGTCCTTGCCAACTTC	GCTGTTGTAGCTTTTGCTCCTCCTATG
*ASL*	AT5G10920	GCAGGACATCTTGATGCGACTACTC	CCTTTTGAGACGCAAACTCCAACTAG
*ASSY*	AT4G24830	GGAAAAGGAAATGACCAGGTTCGG	TCAATAGCATCTTCTCGGCCTTGG
*CARA*	AT3G27740	TGCACGAAGACACTTGCTGA	CTGTGCTCAACACCCCGATA
*CLPD*	AT5G51070	CCGTCCAGAGTTGTTGAACCG	CCACGAGCCTCGACTTCAAGTC
*DIN4*	AT3G13450	GACACTCCTTTCCCTCTAGTGTTCG	ATTCACAGTGGATCTGATTGCATCC
*dOAT*	AT5G46180	TGTCCCCGGTTTCAGCTTAC	AGCCTCAGATCCATCTCGGA
*IMD*	AT5G14200/AT1G31180	CGATGCTTCTCAAGTATGGACTTGG	CCATTTCCTTGCATCCCACCAG
*NAGS2*	AT4G37670	GAAGGAACCCGTGATGCCAGAG	GCTCGCAGTAACTCCTCATCAGTTC
*OTC*	AT1G75330	CAAAGGCAAAGCAAGCTGGA	CGGCTTCATCCTTTTGACCC
*P5CDH*	AT5G62530	GATAGGGACACCAGAGGCTATA	GTAGATGGAGGAAGTTCCCAAC
*P5CS1*	AT2G39800	GGTTGAGACTTGAGGAGAGACAC	CCACTACATAAGCGAGGGTTTCAA
*PII*	AT4G01900	CCATCTTGCCTCGATTTGGTCAC	CAGTAAAGCCGATGAAACTTGCTGG
*PP2A*	AT1G13320	CTGCAAACAATCTGAAGCGTCTTG	CTGGAGCGAGAAGCGATACTG
*PRODH*	AT3G30775	GTGTCGTTCTCGCAACACATAACG	CTTGCTAACATTGAACCCTGCTCTC

### Metabolite analysis

Metabolite extraction and sample preparation was performed as described by Bündig and colleagues [21]. The samples were loaded on a 30 m VF-5ms GC column (Agilent, Santa Clara, USA) according to Lisec and colleagues [22]. Chromatograms were analyzed using Chroma TOF® (Leco, St Joseph, MI, USA). The concentration of each metabolite was determined on the basis of a calibration with standards with known concentrations of each metabolite. Identification as well as quantification is based on the peak height of unique masses for each metabolite (S1 File).

### Quantification of arginine, citrulline, ornithine and urea

The abundance of arginine and ornithine needed to be quantified with an enzymatic assay because the GCMS is unable to distinguish between these two metabolites [23]. Metabolites were extracted as described before [21]. The assay was performed according to Bucci and colleagues [24] with minor modifications. The assay based on four repetitions, one for each of the metabolites arginine, citrulline, ornithine, and urea. Citrulline and urea can be directly stained. Arginine is converted to urea for quantification and ornithine is converted to citrulline.

Speedvac-dried metabolites were dissolved in 80 μl H_2_O and aliquoted to four reaction tubes. The four different assays were conducted in a final volume of 50 μl in 0.4 M triethanolamine (TEA, pH 7.7) in the dark. The reactions were stopped with 50 μl 10% trichloracetic acid. For visualization 200 μl staining solution were added. The staining solution was a mixture of one volume light sensitive diacetylmonoxime [0.4% (w/v) in 7.5% (w/v) NaCl] and two volumes acidic phenazone/ferric ammonium sulfate solution [20 mM phenazone, 5 mM FeNH_4_(SO_4_)_2_ in 25% H_2_SO_4_ and 25% H_2_PO_4_ (85%)]. The mix was incubated at 95°C for 30 min, cooled down in an ice bath for 1 min and centrifuged at 16.000*g* for 1 min. The absorbance of the supernatant was measured at either 460 nm (urea) or 464 nm (citrulline) in an Elisa Reader (Biotek, Winooski, USA).

For the differentiation between citrulline and urea, 1 U urease was added to the TEA followed by 1 h incubation at 25°C. For the quantification of arginine, 1 U Arginase was added to the TEA buffer followed by 1 h incubation at 37°C. Arginase converts arginine to urea and ornithine. Urea was quantified at 460 nm. For the quantification of ornithine, 0.2 U ornithine transcarbamylase (OTC) and 5 mM carbamoyl phosphate dilithium salt were added to the TEA buffer followed by 1 h incubation at 37°C. OTC converts ornithine to citrulline.

### Statistics

Statistical analysis was performed as indicated in the figure legends. Basically, we used a two-tailed Student´s t-test (*p*<0.05) to analyze significance between two data sets. An ANOVA analysis was performed to test both the level of significance of the population mean (*p*<0.05) for urea cycle intermediates and the influence of an additional sugar supply on metabolite levels. For the latter, we additionally used three different mean comparison test (Bonferroni, Tukey and Bonholm) (*p*<0.05) to display the significance levels between the population means per metabolite. All tests gave the same results.

## Results

### The limitation of both CO_2_ and light induces similar changes in metabolites related to carbon starvation

**Fig 2:**
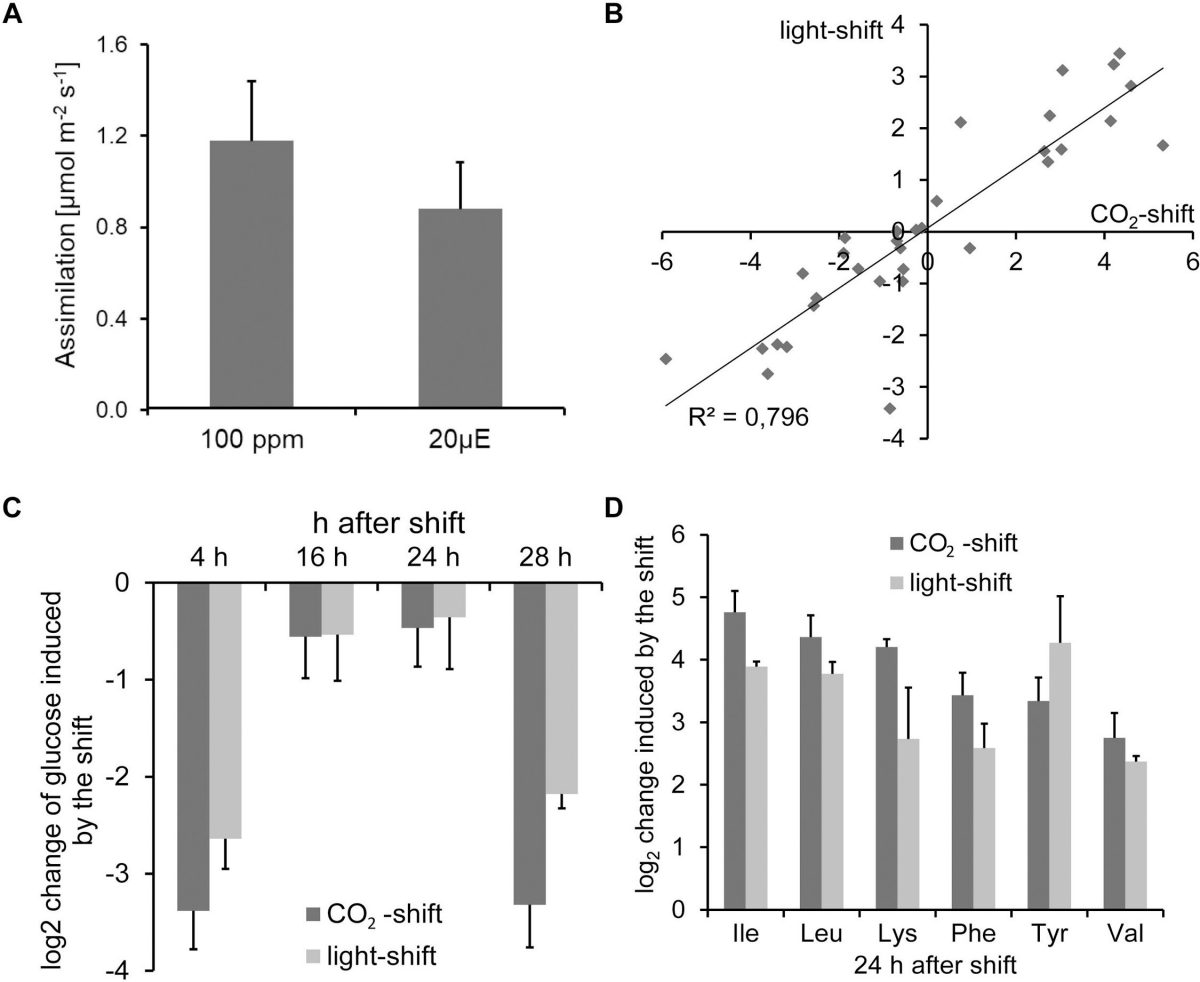
Carbon starvation is induced by both low CO_2_ concentration and low light intensity. (A) Net CO_2_ assimilation of *A*. *thaliana* plants at 100 ppm CO_2_ and 20 μE light intensity, respectively. (B) Correlation in log_2_ changes in metabolite concentrations between plants shifted to either low light or to low CO_2_ concentrations. (C) Reduction of the glucose concentration in response to both low light- and low CO_2_-shift. (D) Log_2_ changes of selected amino acids 24 h after the shifts. Data are the mean of three (light-shift) or five (CO_2_-shift) biological replicates ± SD. The whole data set is given in S1 File.

To investigate the impact of carbon starvation on ornithine accumulation in *Arabidopsis thaliana*, Col-0 plants were subjected to two different conditions. In the first experimental setup, plants were shifted from 400 ppm CO_2_ to 100 ppm CO_2_, in the second setup they were shifted from a light intensity of 150 μE to 20 μE. Gas exchange measurements showed that plants shifted to either low CO_2_ or low light have similar net carboxylation rates of about 1 μmol m^-2^ s^-1^ (Fig 2A). Therefore, the degree of carbon starvation is comparable between the two treatments and effects can be directly compared. The assimiliation rate under control conditions was determined as 8.61 ± 1.02 μmol m^-2^ s^-1^ (S2 File).

In a first experiment, changes in metabolite concentrations in response to both treatments were analyzed by GC-MS. To monitor metabolite changes in a diurnal profile either five (light-shift) or six (CO_2_-shift) time points were selected for harvesting. In total 33 metabolites were accurately determined by the GC-MS analysis (Fig 2B and S1 File). A comparison of the metabolic changes between the two treatments revealed a good correlation (Fig 2B). Both, low CO_2_ and low light caused a downregulation of the synthesis of carbohydrates. The expected reduction in glucose concentrations was observed under both conditions (Fig 2C). Consequently, the glucose concentration was up to eight times lower than under control conditions throughout the day. During the night, the glucose concentration was reduced by approximately 1.5 fold in both treatments. Among the metabolites that showed a strong induction under both conditions the amino acids isoleucine, leucine, lysine, phenylalanine, tyrosine, and valine were induced between 5- and 27-fold 24 h after the shift compared to their respective controls (Fig 2D). These amino acids are known to accumulate in response to sugar starvation induced by prolonged darkness [25–27]. To track the accumulation of these amino acids, we exemplarily measured genes of leucine synthesis and degradation, respectively. We selected isoprolylmalate dehydrogenase (*IMD*) that is involved in leucine synthesis and dark-induced 4 (*DIN4*) as a representative for leucine degradation. Surprisingly, *IMD* was strongly repressed in response to low CO_2_, *DIN4* showed the opposite behavior (S2 File). Unspecific amino acid accumulation might also be caused by protein degradation. Therefore, we measured the expression levels of the protease *CLPD*, which is strongly induced in response to drought [28]. Upon low CO_2_ treatment, *CLPD* expression was 3- to 4-fold increased during the day and early night. However, at the end of the night the expression declined back to the control level (S2 File).

In summary, both CO_2_ and light limiting conditions led to a comparable reduction in sugar contents, and more importantly, they showed similar responses related to carbon starvation.

### Ornithine is exclusively induced in response to low CO_2_ concentrations

**Fig 3:**
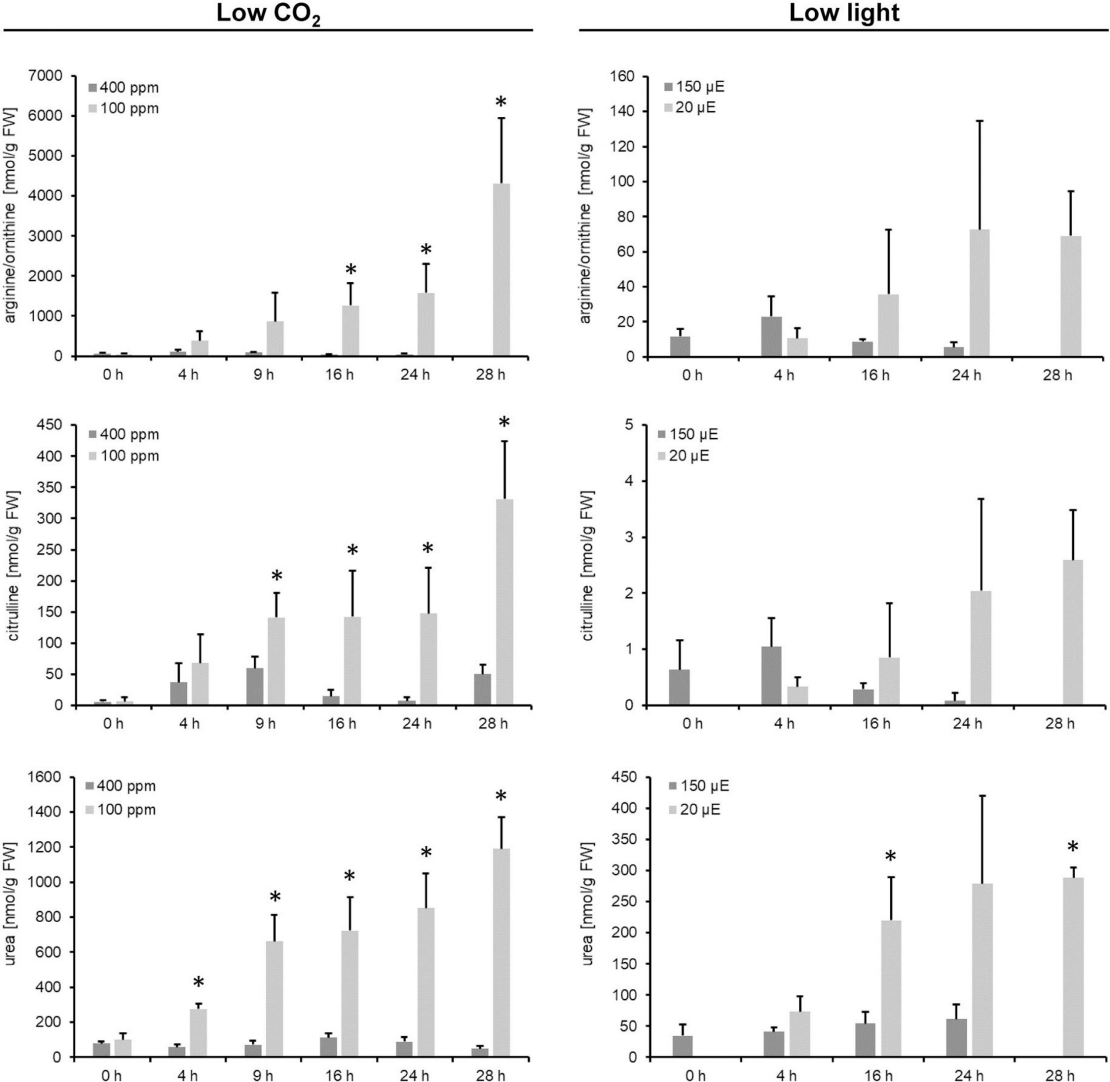
Log_2_ changes induced by either the CO_2_- or the light-shift in metabolites of the urea cycle. Data is obtained by GCMS analysis. Data points represent the mean of three (light-shift) or five (CO_2_-shift) biological replicates ± SD. The whole data set is given in S1 File. Significance was tested according to the two-tailed Student's t-test (* p<0.05). Additionally, the level of significance of the population mean was tested by ANOVA (p<0.05). Results are listed in S1 File.

Despite the common responses of both treatments to the carbon starvation, there were significant differences in metabolites of the urea cycle (Fig 3 and S1 File). While urea cycle metabolites strongly increased in plants under CO_2_ limitation, the changes were much less pronounced for these metabolites in plants grown under low light conditions. A two-way ANOVA test was performed to analyze the effect of both the CO_2_ treatment and the time factor. The CO_2_-treatment significantly affected metabolite levels in response to both low CO_2_ and low light. However, F-values determined for the low CO_2_ populations were 10-fold higher (low CO_2_—arginine/ornithine, F = 79.2; citrulline, 87.9; urea, 441.5; low light—arginine/ornithine, F = 8.0; citrulline, 6.1; urea, 48.3) indicating that low CO_2_ had a much stronger influence than low light has (S1 File). With the exception of Arg/Orn levels (low light), the length of the treatment also significant influenced metabolite accumulation (S1 File).

A limitation of the GC-MS analysis is that some metabolites are converted into a different metabolite upon trimethylsilylation with MSTFA [23]. This is also true for arginine that is converted to ornithine upon trimethylsilylation. Thus, MSTFA-derivatized ornithine and MSTFA-derivatized arginine are indistinguishable in both the retention time and mass spectra [23]. Hence, we determined the concentration of both metabolites by a colorimetric assay.

**Fig 4:**
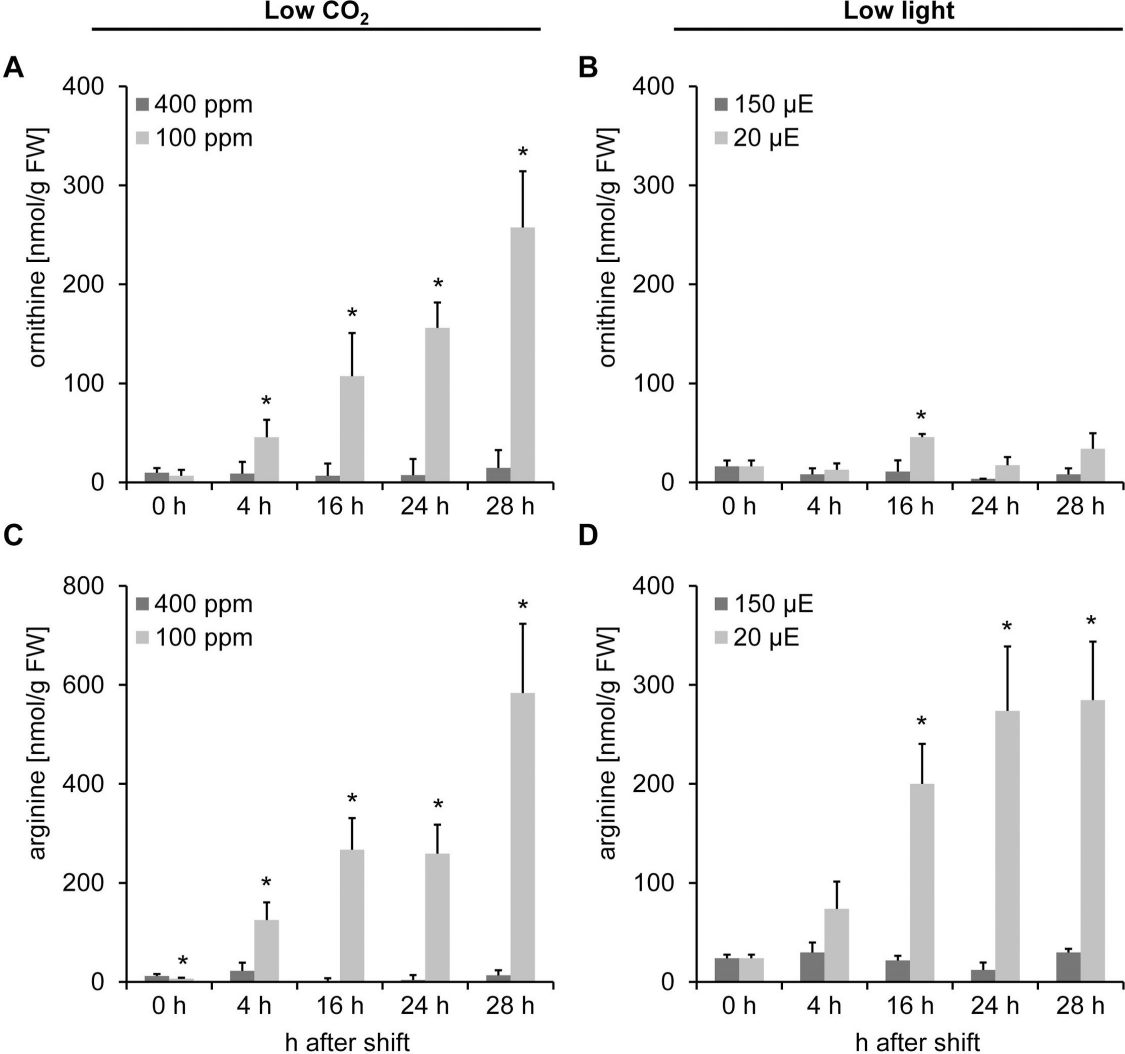
**Ornithine (A, B) accumulates exclusively in response to low CO**_**2**_ **concentrations, arginine (C, D) accumulates in response to both, the CO**_**2**_**- and the light-shift.** Metabolite concentrations were determined by a colometric assay as described in Methods. Data represent the mean of three (light-shift) or five (CO_2_-shift) biological replicates ± SD. Significance was tested according to the two-tailed Student's t-test (* p<0.05).

While ornithine was hardly detected in plants grown under 400 ppm CO_2_, ornithine accumulated in plants shifted to 100 ppm CO_2_ (Fig 4A). After 28 h, the ornithine concentration was 18 times higher in plants shifted to 100 ppm CO_2_ relative to the control plants grown under 400 ppm CO_2_. In contrast, ornithine did not accumulate in response to low light. Its concentration was close to the detection limit (Fig 4B). Ornithine and urea are products of a reaction catalyzed by arginase in which arginine is used as a substrate [8]. In agreement with this, arginine also strongly increased in response to the low CO_2_-shift to a comparable extent (Fig 4C). However, the increase in arginine accumulation in the diurnal course was also observed for plants shifted to low light conditions, but to a much lower extent (Fig 4D).

Thus, the accumulation of ornithine, citrulline and urea is a specific response to the low CO_2_ treatment.

### The accumulation of both ornithine and citrulline is independent from externally supplied sucrose

**Fig 5:**
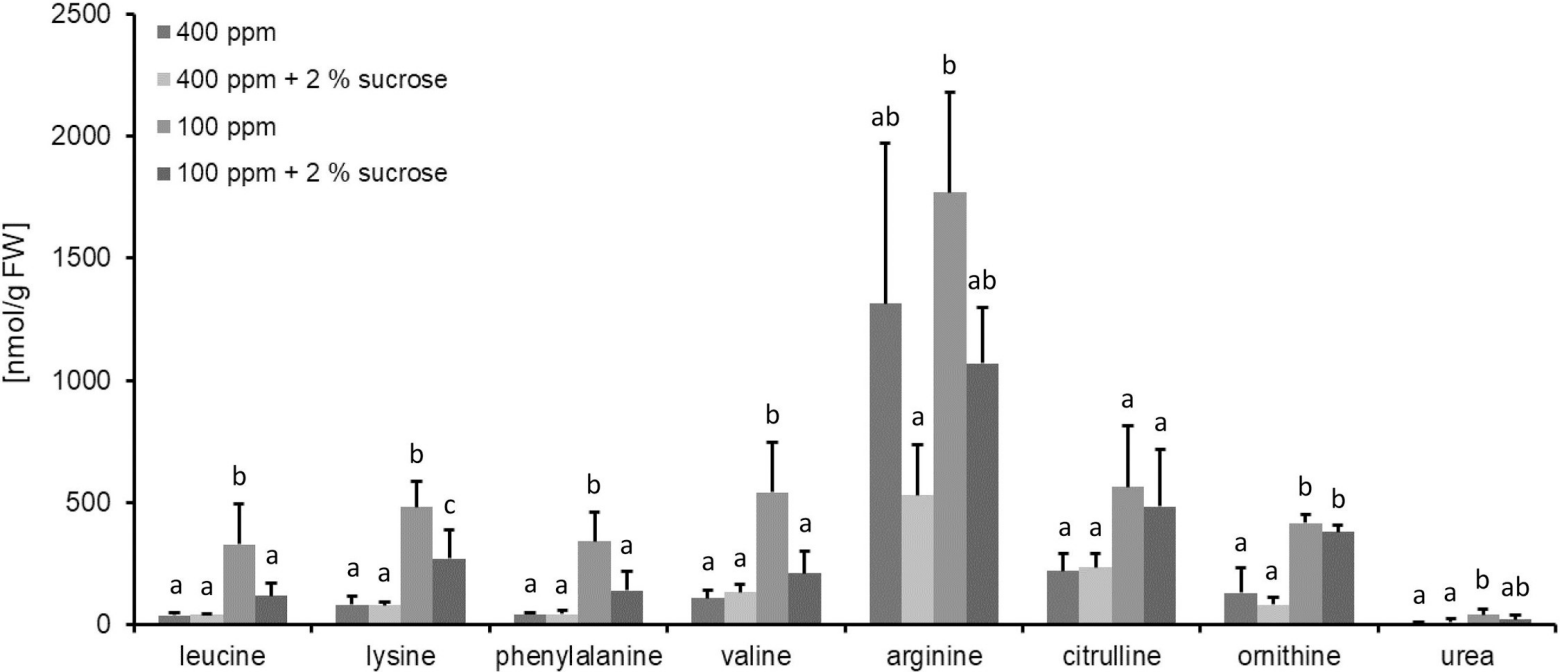
Ornithine accumulation in response to low CO_2_ is independent of sugars. Log_2_ changes of selected amino acids 24 h after the CO_2_-shift. Plants were grown on media with or without 2% sucrose. Data is obtained by GCMS (Leu, Lys, Phe, Val and urea) or by an enzymatic assay (Orn, Cit, and Arg). Data are the mean of five biological replicates ± SD. The whole data set is given in S1 File. The level of significance of the population mean was tested by ANOVA (p<0.05). Mean comparison was performed as described in the Methods section. Identical letters indicate no significant difference between the mean populations. Results are listed in S1 File.

Based on the data above we hypothesized that the accumulation of urea cycle intermediates is independent of carbon starvation, and must be related to a different response. In order to evaluate the impact of externally supplied sugar on the accumulation of amino acids, plants were grown on ½ MS media supplemented with 2% sucrose for three weeks. In a first set of experiments, the impact of low CO_2_ on amino acid accumulation was analyzed for plants grown on ½ MS. As observed for soil grown plants, amino acids accumulated also in response to low CO_2_ concentrations when plants were grown on ½ MS (Fig 5 and S1 File). Again, ornithine, arginine and additionally citrulline were determined by a colorimetric assay. A two-way ANOVA analysis revealed that the CO_2_ concentration had a significant influence on all metabolite levels, while sugar supply only significantly influenced the metabolite levels of leucine, lysine, phenylalanine, valine and arginine (S1 File). Even though arginine metabolite levels were significantly influenced by sugar supply and the low CO_2_-shift, no interplay between the two factors was observed by ANOVA analysis (S1 File). Likewise, metabolite levels of citrulline, ornithine and urea were not affected by sugar supply when simultaneously shifted to low CO_2_ (S1 File).

Thus, the accumulation of urea cycle intermediates in response to low CO_2_ concentrations is independent from the sugar status of the cell.

### Changes in metabolite abundances under abiotic stress and mutants related to both ornithine metabolisms and photorespiration

Having determined metabolite abundances in response to low CO_2_, we wanted to compare the observed changes to metabolite patterns of other stress experiments and mutants. We have chosen metabolite data from three different publications. In photorespiration, oxidative stress in induced by an increased production of hydrogen peroxide by glycolate oxidase in leaf peroxisomes [3]. Oxidative stress is also induced under high light conditions [29]. Here, oxidative stress is caused by an over-reduction of the electron transport chain in leaves. In addition, we chose data from the photorespiratory mutant *ggt1* [30] because low CO_2_ conditions increase photorespiration. In the *ggt1* mutant photorespiration is blocked. Lastly, there were metabolite data available for T-DNA insertion lines of the δ*OAT* gene (*oat1* and *oat3* mutant) [7].

**Fig 6:**
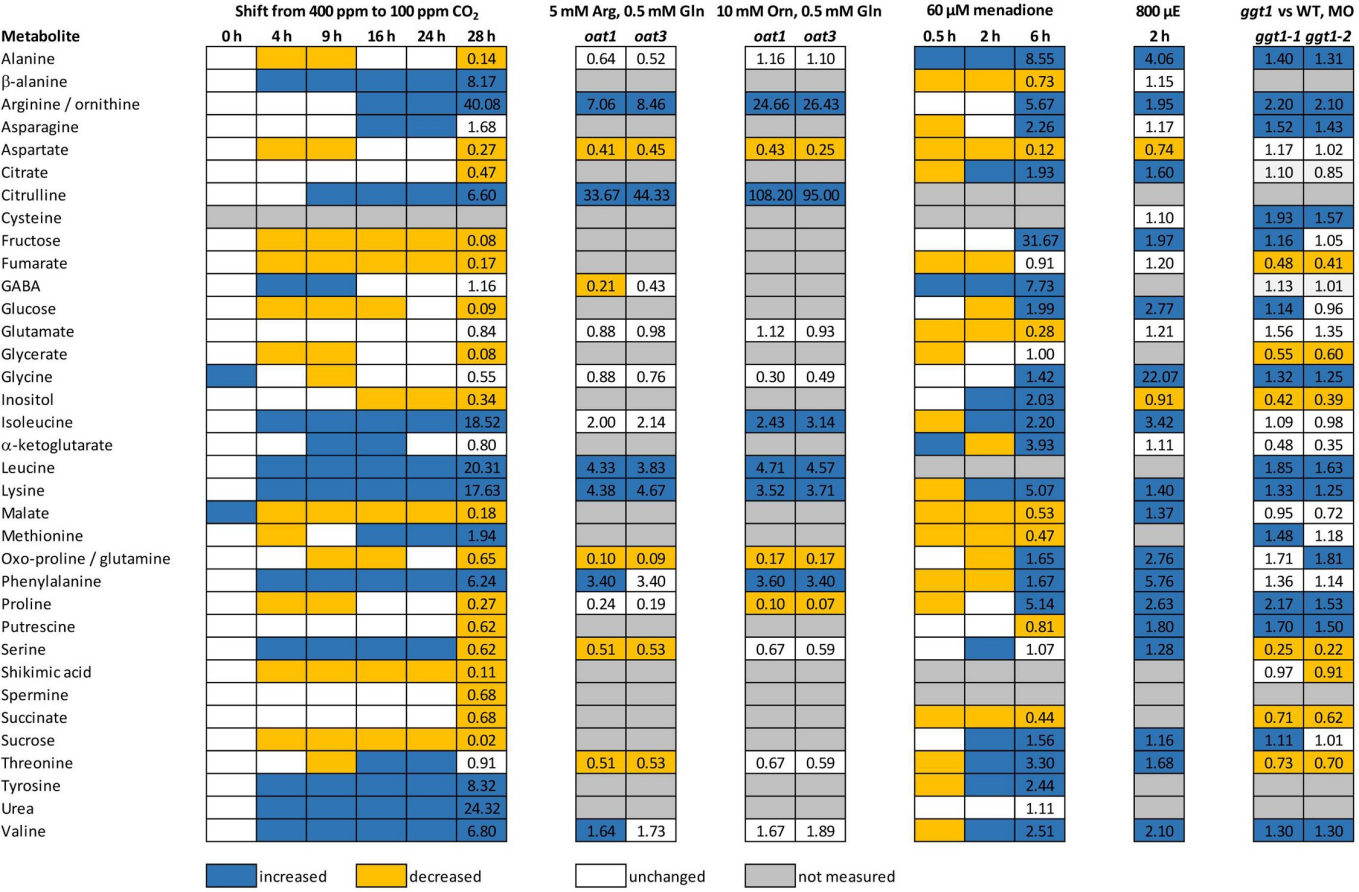
Comparison of metabolite changes in response to different stresses and in different *Arabidopsis* mutants. Data listed were extracted from publications by Funck et al. [7] (*oat* mutants in response to nitrogen limitation), Florez-Sarasa et al. [29]) (high light treatment), and Dellero et al. [30] (*ggt1* mutant). Significant metabolite changes were determined by Student’s t-test (*p*<0.05) in our study and the selected publications, respectively. Numbers in selected columns indicate fold-changes relative to the control conditions. Given that Funck et al. [7], Florez-Sarasa et al. [29] and Dellero et al. [30] provided data for both arginine and ornithine, these fold changes were averaged in order to make them comparable to our GC-MS metabolite data. *oat*: T-DNA insertion in the gene encoding *Arabidopsis* ornithine-δ-aminotransferase, Arg: arginine, Gln: glutamine, Orn: ornithine, h: hours, *ggt1*: T-DNA insertion in the gene encoding *Arabidopsis* glutamate:glyoxylate aminotransferase 1, MOD: middle of the day. Blue: significantly induced, orange: significantly reduced, white: unchanged, grey: not measured.

Fig 6 summarizes changes in metabolite abundances (treatment vs. control) determined in this study and in the studies mentioned above. From our data, it is obvious that most of the metabolites respond only in one direction. A few exceptions like methionine and threonine showed a mixed behavior. For comparing our data with other publications, we chose the time point 28 h after the shift because we expected that the metabolism had reached a steady-state in response to the low CO_2_-shift at this point. In general, it should be mentioned that the comparison with the other sources was limited by the variability of totally determined metabolites. The photorespiratory mutant *ggt1* primarily accumulates all metabolites in the middle of the day (which is the time point the data have been recorded) and, thus, under conditions where photosynthesis and consequently photorespiration are highly active in C3 plants like *Arabidopsis*. However, one third of the metabolites (only amino acids) measured showed common responses when compared to our data. Differences were seen for alanine, aspartate, glycerate and phenylalanine for instance (Fig 6). A 2 h high light treatment only resembled seven changes that were observed in response to low CO_2_. Interestingly, there were major differences in the abundance of both proline and putrescine. While both metabolites increased in response to high light, the abundance was decreased in response to low CO_2_. The decrease in proline was already observed 4 h after the shift to limiting CO_2_ conditions (Fig 6). However, the *oat* mutants qualitatively showed the best common responses when compared to our data.

In order to visualize not only qualitative changes of metabolites between the low CO_2_-shift and different stresses/different *Arabidopsis* mutants, we performed a correlation analysis to quantitatively compare changes of metabolites (S2 File). This means, that we transformed the fold changes in metabolite abundance of both our data and originating from references [7], [29] and [30] into log_2_ fold metabolite changes. In the graphics, each log_2_ fold metabolite change of the low CO_2_-shift (x-axis) was plotted against the respective change in the selected reference (y-axis) (S2 File). The coefficient of determination (Table 2 and S2 File) reflects the regression of all metabolites relative to a perfect correlation of 1. Thus, the closer to 1, the better the correlation between all metabolite changes in response to the CO_2_-shift and the treatment compared to. Pearson´s correlation coefficient (PCC) is a different measure to display the correlations and is often used to describe the strength of co-expression between to genes [31]. PCC can take a value between 0 and 1 in case of a positive correlation. The strength is defined as no correlation (0 ≤ r ≤ 0.2), weak to moderate (0.2 < r < 0.5), distinct (0.5 < r < 0.8) and strong to perfect (0.8 < r < 1.0).

**Table 2:** Coefficient of determination and Pearson´s correlation coefficient of log_2_ metabolite changes between the low CO_2_-shift and in response to different stresses/different *Arabidopsis* mutants.

Stress/mutant, condition	Coefficient of determination (R^2^)	Pearson´s correlation coefficient	Source of correlated metabolite data
*oat1*, 5 mM Arg, 0.5 mM Gln	0.5708	0.756	[5]
*oat3*, 5 mM Arg, 0.5 mM Gln	0.5927	0.770	[5]
*oat1*, 10 mM Orn, 0.5 mM Gln	0.5383	0.734	[5]
*oat3*, 10 mM Orn, 0.5 mM Gln	0.5862	0.766	[5]
800 μE, 2 h	0.0104	0.102	[29]
*ggt1-1*, MOD	0.1194	0.345	[30]
*ggt1-2*, MOD	0.1327	0.364	[30]

Visualization of correlation is given in S2 File. *oat*: T-DNA insertion in the gene encoding *Arabidopsis* ornithine-δ-aminotransferase, Arg: arginine, Gln: glutamine, Orn: ornithine, h: hours, *ggt1*: T-DNA insertion in the gene encoding *Arabidopsis* glutamate:glyoxylate aminotransferase 1, MOD: middle of the day.

Table 2 summarizes the coefficient of determination and PCC for log_2_ fold metabolite changes determined on the basis of the fold changes indicated in Fig 6 and the correlation analysis in S2 File. Both the coefficients of determination and PCC calculated for the *oat* mutants indicate a distinct correlation between the metabolite changes between the low CO_2_-shift and under 5 mM arginine/0.5 mM glutamine and 10 mM ornithine/0.5 mM glutamine feeding of the *oat* mutants, respectively. No correlation was observed when the log_2_ metabolite changes of the low CO_2_-shift were correlated to light induced stress, a weak to moderate correlation when correlated to the *ggt1* mutants (Table 2).

This means that the metabolite changes observed in response to the low CO_2_-shift most likely resemble those observed in the *oat* mutants.

### Source of ornithine and citrulline accumulation

We wished to analyze the specific accumulation of both ornithine and citrulline in more detail and followed two different strategies. First, we determined changes in the transcription of genes either involved in ornithine synthesis/degradation or functioning in the connected pathways of polyamine and proline synthesis. Second, we evaluated changes in metabolite abundance in proline and the two polyamines putrescine and spermine. Both proline and polyamines are important metabolites in abiotic stress responses [32–34]. Thus, the accumulation of ornithine might simultaneously led to their accumulation in response to low CO_2_.

According to Slocum [7] ornithine can enzymatically be produced by two different reactions. Firstly, ornithine can be produced from glutamate via various steps in the chloroplast. Secondly, arginine can be directly converted to ornithine and urea by arginine amidohydrolase (ARGAH) in mitochondria. In *Arabidopsis*, two ARGAH isoforms *ARGAH1* and *ARGAH2* exist [9]. The analysis of the expression patterns of both genes revealed that *ARGAH2* is the major isoform expressed in leaf tissue, while *ARGAH1* is predominantly expressed in reproductive tissues [9]. As described in the introduction, ornithine can be converted into pyrroline-5-carboxylate (P5C) by ornithine-δ-aminotransferase (δOAT) [35]. Furthermore, ornithine can be metabolized to arginine by a three-step reaction sequentially catalyzed by ornithine transcarbamylase (OTC), argininosuccinatesynthetase (ASSY) and argininosuccinatelyase (ASL) in chloroplasts. Here, the reaction catalyzed by OTC yields citrulline. Citrulline can also be produced by nitric oxide synthase (NOS) from arginine [36].

**Fig 7:**
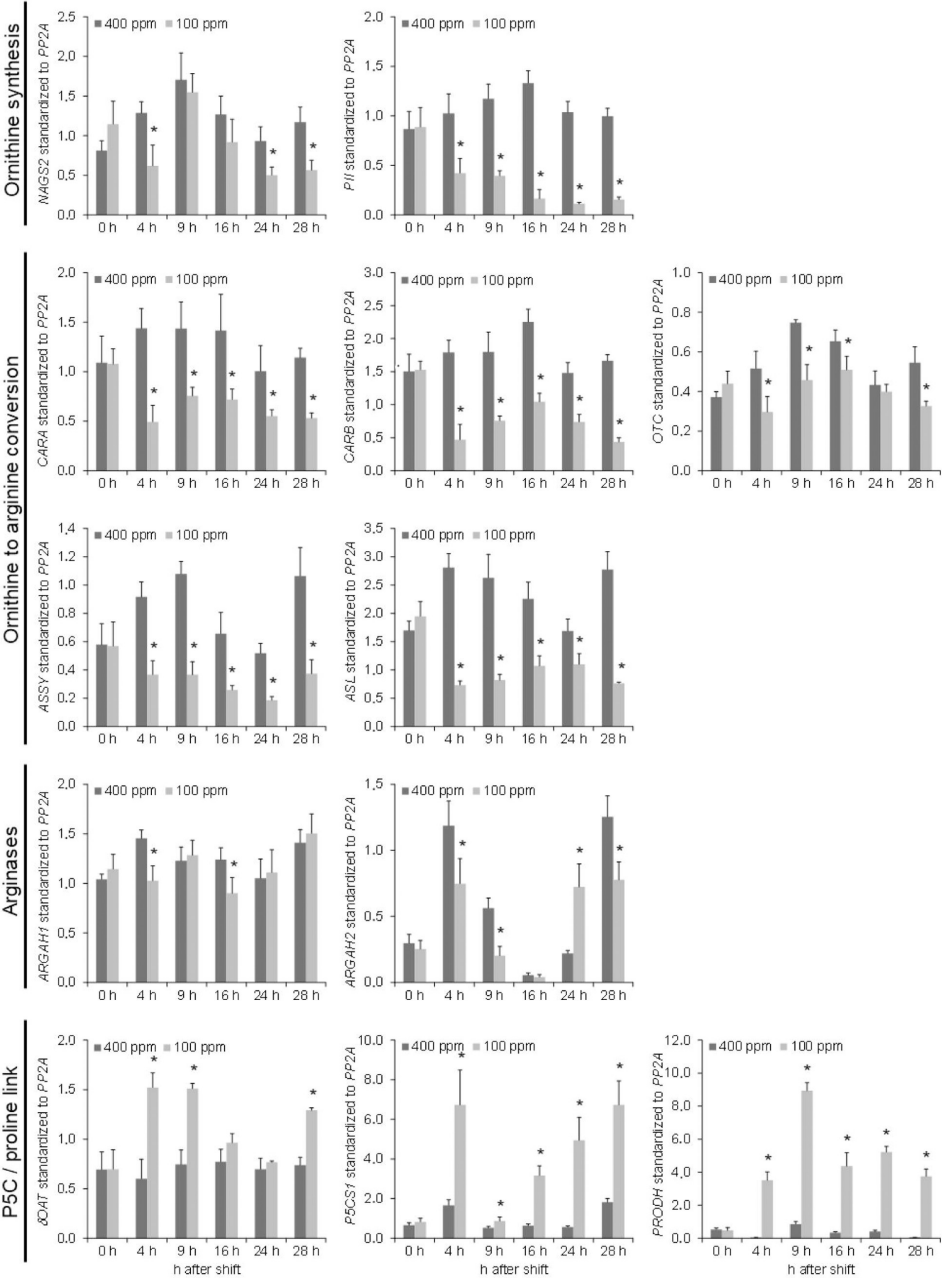
Transcript levels of genes encoding either urea cycle enzymes or enzymes in linked pathways determined at 400 ppm and 100 ppm CO_2_ and standardized to *PP2A*. Data are the mean of five biological replicates ± SD. Significance was tested according to the two-tailed Student's t-test (* p<0.05). Values were expressed as log_2_ ratios to allow this test. *ARGAH*, arginine amidohydrolase; *ASL*, argininosuccinate lyase; *ASSY*, argininosuccinate synthetase; *CARA*, carbamoyl phosphate synthetase A;*CARB*, carbamoylphosphate synthetase B; *NAGS2*, N-acetyl-L-glutamate synthase 2; *δOAT*, ornithine-δ-aminotransferase; *OTC*, ornithine transcarbamylase; *P5CS1*, pyrroline-5-carboxylate synthetase; *PRODH*, proline dehydrogenase.

The qPCR data showed that all tested genes encoding chloroplast located enzymes involved in either the conversion of ornithine to arginine or the regulation of ornithine synthesis from glutamate were decreased in response to low CO_2_ (Fig 7). The decrease in transcripts for *CARA/B*, *OTC*, *ASSY*, *ASL* and *PII* was already observed 4 h after shifting the plants to 100 ppm CO_2_ and generally remained constant throughout the diurnal rhythm. In the case of *PII*, the repression of transcript levels steadily declined throughout the day to levels of 10% of the initial value at the beginning of the shift. However, the expression of the genes encoding enzymes involved in the conversion of ornithine to arginine was significantly lowered in a range of 30 and 75%. *OTC* transcript levels significantly decreased in the light phase, but recovered to control levels at the end of the night.

To analyze whether P5C formation from either ornithine conversion or proline degradation was affected, we measured the transcript levels of δ*OAT*, pyrroline-5-carboxylate synthase (*P5CS1*) and proline dehydrogenase (*PRODH*), respectively. Quantitative qPCR revealed that δOAT is upregulated by approximately 2-fold in response to the CO_2_-shift only in the light phase of the day (Fig 7). Additionally, *P5CS1* and *PRODH* were strongly upregulated throughout the day. However, the two genes showed distinct expression peaks with *P5CS1* being highly expressed during the day and *PRODH* being highly expressed shortly after offset of illumination. The expression levels at later time points in the night were comparable between the two genes when standardized to *PP2A* that is encoding for protein phosphatase 2A.

*ARGAH1* was both stably expressed in the diurnal rhythm and hardly responding to low CO_2_ concentrations on transcript level (Fig 7). Conversely, expression of *ARGAH2* showed a distinct diurnal rhythm with a peak at midday but a minimum expression in the middle of the night. The shift to low CO_2_ concentration had a negative impact on maximum transcript abundance both in the light and early night, while expression was boosted in the second half of the night.

In summary, genes encoding enzymes in P5C production were strongly induced, while genes encoding enzymes both in ornithine production from glutamate and in ornithine conversion to arginine in the chloroplast were repressed throughout the day. δ*OAT* expression was only significantly induced in the light and shortly after the offset of light (Fig 7).

**Fig 8:**
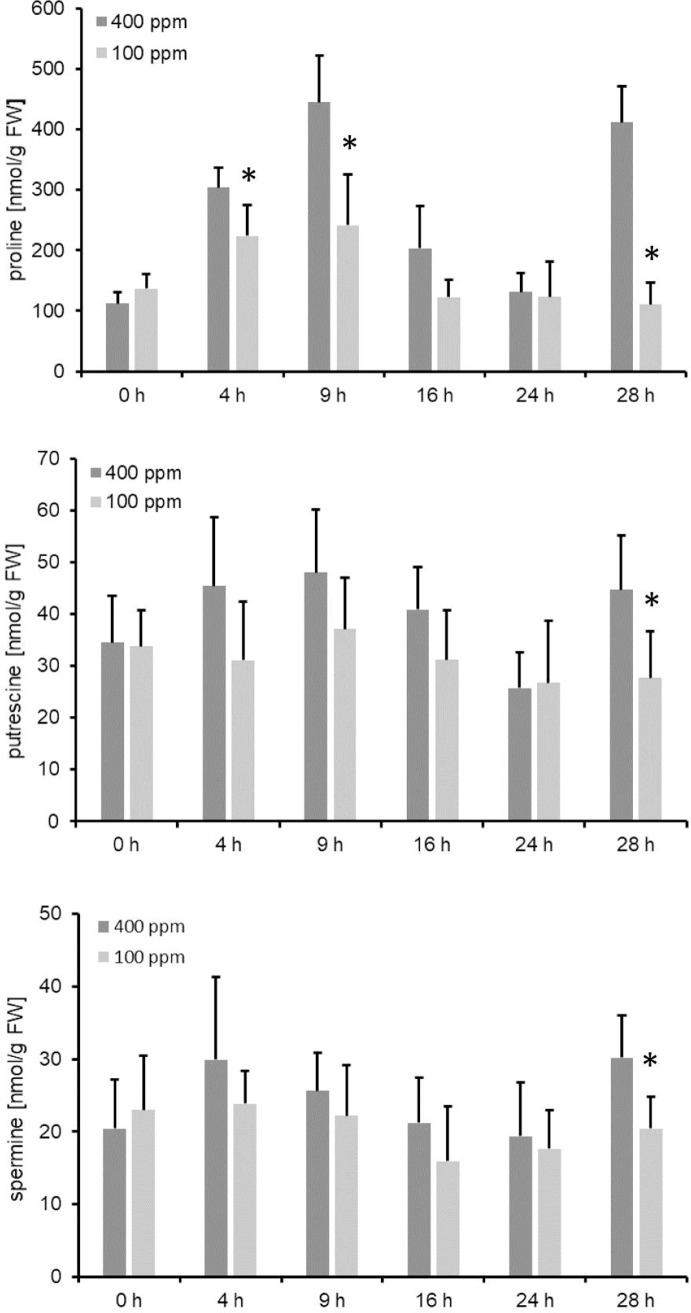
Accumulation of proline and the polyamines putrescine and spermine in response to low CO_2_. Metabolite abundances were obtained by GCMS. Data are the mean of five biological replicates ± SD. The whole data set is given in S1 File. Significance was tested according to the two-tailed Student's t-test (* p<0.05).

In accordance with a stronger induction of *PRODH* gene expression compared to *P5CS*, we observed a decrease in proline accumulation in response to CO_2_ limitation (Fig 8). However, despite the strong accumulation of ornithine, citrulline and arginine the metabolite abundance of the polyamines putrescine and spermine was unchanged within the first 24 h after the shift, and decreased in the following (Fig 8).

## Discussion

**Fig 9:**
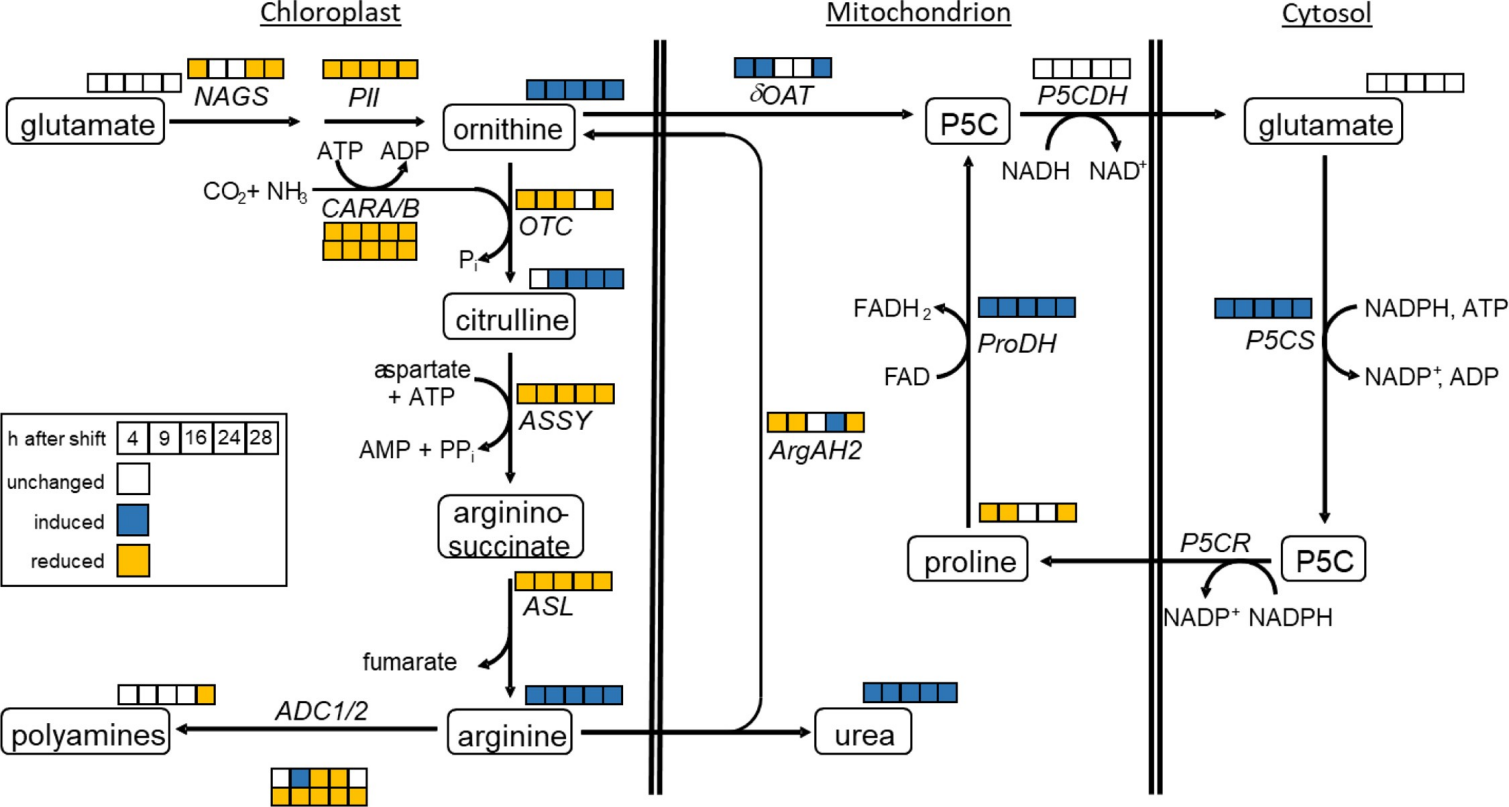
Integration of metabolite and transcript data of ornithine-linked pathways. The squares indicate changes on both transcription and metabolic level in response to a CO_2_-shift from 400 ppm to 100 ppm (Student´s t-test, *p*<0.05). ADC1/2, arginine decarboxylase 1 and 2; ArgAH, arginine amidohydrolase; ASL, argininosuccinate lyase; ASSY, argininosuccinate synthetase; CARA, carbamoyl phosphate synthetase A; CARB, carbamoyl phosphate synthetase B; NAGS, N-acetyl-L-glutamate synthase; δOAT, ornithine-δ-aminotransferase; OTC, ornithine transcarbamylase; PII, PII protein; P5CDH, pyrroline-5-carboxylate dehydrogenase; P5CS, pyrroline-5-carboxylate synthetase; P5CR, pyrroline-5-carboxylate reductase; PRODH, proline dehydrogenase. Blue: significantly induced, orange: significantly reduced, white: unchanged.

In this manuscript, we provide evidence that both ornithine and citrulline accumulation under low CO_2_ is distinct from metabolic changes that can be attributed to energy depletion (Figs 3, 4 and 5). For a better overview on the regulation of ornithine and citrulline metabolic pathways, we summarized transcript and metabolite data in an additional figure, in which the significant changes were displayed throughout the entire time course of the shift experiment (Fig 9). The figure points out that even though ornithine, citrulline, arginine and urea are accumulating, the enzymes within the pathways were predominantly downregulated. Metabolite levels of glutamate and the two polyamines putrescine and spermine were unchanged (Fig 8 and S1 File). Genes encoding enzyme involved in proline synthesis/degradation were upregulated (Fig 7), and proline levels were unchanged throughout the first 24 h after the shift (Fig 8). Thereafter, proline degradation predominates. *ARGAH2* expression was the most inconsistent upon all genes tested (Fig 7). δ*OAT* transcription was initially induced, unchanged during the night, but induced again in the light (Figs 7 and 9).

### Low CO_2_ and low light conditions mediate common responses of carbon starvation

Depletion of the carbohydrates glucose, fructose, and sucrose was detected for both the low CO_2_- and the low light-shift (Fig 2C and S1 File). Additionally, accumulation of several proteinogenic amino acids like leucine, isoleucine, phenylalanine, and valine was observed (Fig 2D) [6]. These responses are already known in plants that face energy depletion as a consequence of light limitation [25–27]. Caldana and colleagues [26] used the combination of transriptomic and metabolomic data to study physiological changes in plants in response to several abiotic stresses. They described the apparent paradoxon that branched chain amino acids and especially leucine accumulate under carbon limitation although amino acid degradation is induced and synthesis is inhibited on transcriptional level, respectively. In this case, the accumulation of amino acids was a matter of the enhanced expression of proteases. Similarly, Brouquisse and colleagues [37] observed nutrient mobilization mechanisms like protein degradation in leaves of dark-stressed maize seedlings.

Amino acids are an excellent energy source [38]. Here, energy obtained especially from hydrophobic amino acids reach yields similar to glucose. Consequently, protein and continuing amino acid breakdown can compensate for a lack of carbohydrates. In this study, the induction of leucine degradation represented by *DIN4* and repression of leucine synthesis represented by *IMD* indicated that similar processes were induced under low CO_2_, low light and in extended darkness (S2 File) [26]. Along with this, the enhanced expression of the protease *CLPD* (S2 File) fits well to the observation that low CO_2_ induces protein degradation (Fig 2D).

Some metabolites like alanine, asparagine, aspartate and glycine show differences between the two treatments. All these changes can be attributed to the photorespiratory C2 cycle. Glycine, which is part of this cycle, is known to be responsive to the photorespiratory rate [39]. At low light, photorespiration is low as is the level of glycine. Alanine and aspartate can act as amino donors for the transamination of glyoxylate, which explains their low concentration at low CO_2_ [40]. Asparagine serves as terminal amino group acceptor during degradation of amino acids [41], which explains its accumulation at low light. At low CO_2_, aspartate concentration is low, and thus fails to be converted to asparagine. Consequently, the behavior of both ornithine and citrulline is an exception, as their accumulation can neither be linked to low carbon gain nor to the photorespiratory C2 cycle.

### Accumulation of urea cycle intermediates upon CO_2_ limitation

Fig 9 points out that the metabolic pathways of both arginine and proline were differently regulated in response to low CO_2_. While genes encoding enzymes in arginine synthesis were downregulated by the treatment, transcription of genes involved in proline synthesis and degradation were induced (Fig 7). δOAT connects both pathway by transferring the δ-amino group of ornithine to α-ketoglutarate or related α-keto acids, thus, generating L-glutamate-5-semialdehyde (GSA) and glutamate. GSA spontaneously converts into the cyclic P5C, a central metabolite in proline synthesis and degradation [42]. In the following, we would like to discuss the accumulation of urea cycle intermediates, especially the accumulation of ornithine and citrulline, based on the transcriptional and metabolic data of the individual pathways.

*Arabidopsis* plants shifted to low CO_2_ conditions showed a significant increase in ornithine and citrulline, intermediates of the biosynthetic pathway of arginine (Figs 3 and 4). In contrast, transcripts of genes encoding enzymes in this pathway were predominantly downregulated (Fig 7). Arginine biosynthesis is regulated by end product inhibition of the enzyme N-acetyl glutamate kinase (NAGK) [11]. Because the metabolite levels of arginine accumulated in response to low CO_2_, we assume the pathway to be systematically downregulated by feedback inhibition. Hence, genes encoding enzymes in arginine biosynthesis were repressed (Fig 7). This theory is further supported by the decrease of *PII* transcript in the course of the treatment (Fig 7). PII can sense 2-oxoglutarate and was shown to control arginine biosynthesis by mitigating the inhibitory effect of arginine on NAGK activity [13]. Consequently, a downregulation in genes encoding enzymes within the pathway will simultaneously led to the accumulation of intermediates.

Arginine has a low C/N ratio and, thus, serves as a major nitrogen storage compound in plants. Arginine accumulation was also reported under stress. Even though the pathway of arginine biosynthesis was downregulated (Figs 7 and 9), we still observed further accumulation of arginine throughout the time course (Fig 4). However, to date there is no reasonable explanation for Arg accumulation in response to low CO_2_. Together with ornithine, arginine serves as a precursor for polyamine (PA) synthesis. Polyamines play a role in tolerance against high salinity and osmotic stress [43]. They function as antioxidants and scavenge free radicals. Moreover, they have an effect on several antioxidant enzyme activities like glutathione reductase and superoxide dismutase [43]. Hence, a major role of PAs is to counterbalance ROS production in response to stress [44,45]. Thus, it is quite surprising that the metabolite abundance of both putrescine and spermine was not elevated (Fig 8) even though i) its direct precursors, arginine and ornithine, accumulated (Figs 3 and 4) and ii) the increase photorespiration led to an increase in the cellular concentration of hydrogen peroxide.

A different explanation for the accumulation of ornithine and citrulline is directly linked to an increase in ammonium assimilation that is stimulated by an increase in photorespiration [46,47]. Taira and colleagues [48] proposed a direct link between ornithine and photorespiration. The authors suggested citrulline to be a shuttle for photorespiratory NH_3_ and CO_2_ from mitochondria to chloroplasts. During photorespiration, CO_2_ and NH_3_ are released from glycine in the mitochondria. Mitochondrial glutamine synthetase can fix ammonia and produce glutamine. Subsequently, glutamine, ATP and CO_2_ are used to produce carbamoylphosphate, which then converts ornithine into citrulline. If a citrulline-ornithine shuttle exists, a stoichiometry between ornithine and citrulline accumulation could be expected. Our data indicated no perfect stoichiometry. The underlying data of Fig 4 gave ratios of ornithine to citrulline between 1.5- and 4.5 fold throughout the time course. Igarashi and colleagues [49] showed that an increase in citrulline is also strongly correlated with glutamate:glyoxylate aminotransferase (GGAT1) mRNA levels and GGAT activity, respectively. The accumulation of citrulline was light dependent and repressed by high CO_2_ concentrations. The authors linked these results to an enhanced photorespiratory flux. Light dependent accumulation of citrulline was also detected under control conditions in our experiments (Fig 3). However, citrulline accumulation was more intense under low CO_2_ and levels did not decline back to the night levels observed under control conditions in the second half of the night, but stayed on the stress level that was reached shortly after the offset of light (Fig 7). Citrulline is a very effective scavenger of hydroxyl radicals, even more effective than mannitol and proline [50]. Accumulation of citrulline was also observed under drought and salt stress in watermelon [50–52].

When discussing ornithine and citrulline as alternative nitrogen sinks following increased photorespiration as a consequence of low CO_2_ it has to be kept in minde that the pool sizes of both metabolites have not the capacity to serve as a sole sink of the nitrogen produced in the C2 cycle. However, ornithine and citrulline are found among the most highly concentrated metabolites after the CO_2_ shift (Figs 3 and 4 and S1 File). As such, they might play a role as an alternative sink for excess nitrogen.

Remarkably, the high rate of photorespiration, and thus the increased consumption of glutamate by GGAT1, did not cause any changes in the metabolite level of glutamate (Fig 9 and S1 File). This phenomenon was observed before in *oat* mutants, under nutrient and environmental stress [7,53–55]. Thus, keeping glutamate levels constant is supposed to be an important feature for cell homeostasis under carbon limitation.

### Simultaneous upregulation of proline biosynthesis and degradation

In *Arabidopsis*, proline plays an important role in stress tolerance and accumulates in response to various stresses [32,56,57]. However, low CO_2_ treatment did not lead to an increase in proline, but rather to a decrease (Fig 8). Our data indicated that the decrease in proline can be attributed to the simultaneous activation of P5CS and PRODH (Fig 7). So far, a reciprocal relationship was reported for the two enzymes [58,59]. Typically, an increase in proline is caused by the inactivation of *PRODH* rather than by an induction of *P5CS* [60–63]. In contrast, *PRODH* is induced under stress release that ultimately leads to proline degradation [61,63,64]. What could be the reason for degrading proline in response to low CO_2_? One explanation is that the breakdown of proline back to P5C in mitochondria feeds electrons either directly or indirectly into the electron transport chain via ubiquinone [65], and thus compensates for carbon limitation. Regarding glutamate homeostasis, it is also likely that proline is degraded to produce glutamate that is either used by GGAT1 in the peroxisome during photorespiration or used to balance glutamate homeostasis in the plant cell.

### Role of δOAT under low CO_2_

Fig 9 shows that the accumulation of urea cycle intermediates in response to low CO_2_ resembled those observed in *oat* mutants under N-limiting conditions [7]. From their results, the authors proposed that δOAT functions as an essential exit route for nitrogen from the urea cycle. This exit route produces GSA/P5C in the first instance, glutamate in the second [42]. Thus, δOAT activity has the capacity to add into proline biosynthesis positively. δOAT function is still discussed controversially. Some publications support a function of δOAT in proline biosynthesis in response to various abiotic stresses [35,66–69]. Others claim little contribution of the ornithine pathway to proline accumulation [7,70], some only under severe stress conditions [71] or during recovery from severe stress [72–75]. Whatever the role of δOAT in response to low CO_2_ conditions, we did not observe an increase in proline accumulation but rather a decrease (Fig 8). Here, the simultaneous increase in proline biosynthesis and degradation (Fig 7) made it difficult to calculate the impact of δOAT. However, overexpression and a knockdown of *δOAT* led to a decrease and increase in ornithine accumulation in response to low CO_2_, respectively (S2 File). Hence, *Arabidopsis* wild type plants actively redirect ornithine into the proline synthesis/degradation cycle under carbon limitation as indicated by an increase in *δOAT* expression (Fig 7).

The remarkable similarity in the accumulation of urea cycle intermediates reported in *oat* mutants by Funck et al. [7] and our data (Fig 6) tempted us to compare parameters like growth conditions, N- and C-supply and *δOAT* expression between the two experimental setups. Table 3 provides this information. While *δOAT* expression is lost in each of the two *oat* mutants *oat1* and *oat3* [7], *δOAT* expression is induced in response to low CO_2_ (Fig 7). The growth conditions between the two experimental setups were quite similar (Table 3). Both studies used short day conditions under moderate light intensities. Experiments performed by Funck and colleagues [7] focused on nitrogen limitation and external supply of various nitrogen sources, while our experiment was done under CO_2_ limitations. Thus, the similarity between the two studies is an altered/imbalanced C/N ratio. The data by Funck et al. [7] clearly demonstrated that *δOAT* expression is needed to mobilize nitrogen from the urea cycle. Because *δOAT* is not downregulated in our experiment, we assume this route to be a possible exit for nitrogen particularily stored in form of ornithine and citrulline during photorespiration. However, the observed induction of δOAT activity in response to low CO_2_ could not counteract ornithine accumulation.

**Table 3:** Comparison of the experimental setup and parameters in Funck et al. [5] and our study.

Parameter	Funck et al. 2008	This study
δOAT expression	Knockout	Gene induced under light
Gowth conditions	MS medium, 110 μE, 9 h light, 22°C	Soil, 150 μE, 8 h light/ 16 h dark, 22°C/20°C; MS medium, 150 μE, 8 h light/ 16 h dark, 22°C/20°C
CO_2_ supply	Ambient	Low (100 ppm)
N-supply	5 mM arginine + 0.5 mM glutamine; 10 mM ornithine + 0.5 mM glutamine	High due to increased photorespiration
Urea cycle intermediates	Accumulation in *oat* mutants under the N-supply given above; arginine and ornithine cannot be used as a sole nitrogen source in the absence of glutamine; urea can be metabolized without any difficulty	Accumulation under low CO_2_ conditions; alternative sink of nitrogen produced by photorespiration?
Proposed/possible function of δOAT	Essential exit rout of nitrogen from urea cycle under physiological conditions	Possible function in glutamate homeostasis and/or production of glutamate for photorespiration

## Conclusion

The present study shows that urea cycle intermediates accumulate independently of the sugar status of the cell under low CO_2_ conditions. This points out that there must be other signals impacting on the C/N balance of the cells under CO_2_ limitation. There is evidence that both guard cells and mesophyll cells can sense the CO_2_ concentration in mediating stomatal movements [76]. As a starting point, future experiments could focus on mutant defective in CO_2_ sensing like the *ht1-2* (*high leaf temperature 1 mutant number 2*) mutant which is defective in HT1 kinase activity and shows a constitutive high CO_2_ response and, thus, a low stomatal conductance [77]. Interestingly, low CO_2_ did not led to an accumulation of polyamines and proline, metabolites generally induced in response to abiotic stress, but to an accumulation of the urea cycle intermediates ornithine and citrulline maybe as an alternative sink of nitrogen produced during photorespiration. Future experiments with knockdown and overexpressing *oat* mutants might uncover the importance of ornithine in the recovery process from short and long term low CO_2_ conditions.

## Supporting information

S1 File Supporting tables. Table A: Parameters that are used for both identification and quantification of metabolites. The Fiehn retention-index was calculated by comparing the retention time of each metabolite with the retention time of fatty acid methyl esters (FAMEs). The indicated masses were checked for a linear correlation before they were was used for quantification of the metabolite. Table B: Metabolic response of *A*. *thaliana* plants shifted from 400 ppm to 100 ppm CO_2_. Table C: Metabolic response of *A*. *thaliana* plants shifted from 150 μE to 20 μE light intensity. Plants were grown in soil under short-day conditions (8/16 h day/night cycle) at a light intensity of 150 μE and 400 ppm CO_2_. After five weeks, half of the plants were shifted to a light intensity of 20 μE before onset of illumination. Whole rosettes were harvested directly after the shift, 30 min before onset of illumination (0 h), at midday (4 h), 1 h after offset of illumination (9 h), at the middle of the night (16 h), 30 min before onset of illumination of the following day (24 h), and at midday of the following day (28 h). Data represent the mean of three biological replicates. Significance was tested according to the two-tailed Student's t-test (* p<0,05; ** p<0.01). Table D: Results two-way ANOVA (Fig 3). Table E: Metabolic response of *A*. *thaliana* plants growing on sucrose supplemented media after a shift from 400 ppm to 100 ppm CO_2_. Plants were grown on 1/2 MS under shortday conditions (8/16 h day/night cycle) at a light intensity of 150 μE and 400 ppm CO_2_. The media of indicated plants was supplemented with 2% sucrose. After three weeks, half of the plants were shifted to 100 ppm CO_2_ before onset of illumination. Whole rosettes were harvested 30 min before onset of illumination of the following day (24 h), and at midday of the following day (28 h). Data represent the mean of five biological replicates. Significance was tested according to the two-tailed Student's t-test with p<0.05 between the CO_2_ condition and the respective sugar treatment only. Table F: Results two-way ANOVA per metabolite (Fig 5).
(XLSX)

S2 File Supporting figures. Fig A: CO_2_ assimilation of 5-weeks-old *Arabidopsis* plants. Net CO_2_ assimilation was recorded under ambient CO_2_ conditions (400 ppm) and light saturating conditions (500 μE). Fig B: Transcript levels of selected genes of leucine synthesis (*IMD*) and degradation (*DIN4*), protein degradation (*CLPD*), and polyamine synthesis (*ADC1*, *ADC2*) at 400 ppm and 100 ppm CO_2_ standardized to *PP2A*. Data represent the mean of five biological replicates ± SD. Significance was tested according to the two-tailed Student's t-test; values were expressed as log_2_ ratios to allow this test (* p<0.05) DIN4, dark-induced 4; IMD, isopropylmalate dehydrogenase; CLPD, caseinolytic protease D; ADC, arginine decarboxylase. Fig C: Correlation of log_2_ metabolite changes between the low CO_2_-shift and in response to different stresses/different *Arabidopsis* mutants. The fold changes are given in Fig 6. Fig D: Concentration of ornithine in knockdown (KD) and overexpression (OE) lines of *δOAT* after a shift from 400 ppm to 100 ppm CO_2_ relative to azygous plants. Plant material was harvested 24 h after shifting the plants to low CO_2_ concentrations. Data are the mean of three (KD) or two (OE) biological replicates ± SD. Grey line: wild type *δOAT* level.
(PDF)
