# Supplementary material for: Low CO2 induces urea cycle intermediate accumulation in Arabidopsis thaliana
Source: PLoS One. 2019 Jan 16;14(1):e0210342. doi: 10.1371/journal.pone.0210342 (PMC6334940; doi:10.1371/journal.pone.0210342)
Supplement: S2 File — Fig A: CO2 assimilation of 5-weeks-old Arabidopsis plants. Net CO2 assimilation was recorded under ambient CO2 conditions (400 ppm) and light saturating conditions (500 μE). Fig B: Transcript levels of selected genes of leucine synthesis (IMD) and degradation (DIN4), protein degradation (CLPD), and polyamine synthesis (ADC1, ADC2) at 400 ppm and 100 ppm CO2 standardized to PP2A. Data represent the mean of five biological replicates ± SD. Significance was tested according to the two-tailed Student's t-test; values were expressed as log2 ratios to allow this test (* p<0.05) DIN4, dark-induced 4; IMD, isopropylmalate dehydrogenase; CLPD, caseinolytic protease D; ADC, arginine decarboxylase. Fig C: Correlation of log2 metabolite changes between the low CO2-shift and in response to different stresses/different Arabidopsis mutants. The fold changes are given in Fig 6. Fig D: Concentration of ornithine in knockdown (KD) and overexpression (OE) lines of δOAT after a shift from 400 ppm to 100 ppm CO2 relative to azygous plants. Plant material was harvested 24 h after shifting the plants to low CO2 concentrations. Data are the mean of three (KD) or two (OE) biological replicates ± SD. Grey line: wild type δOAT level. (PDF) [file pone.0210342.s002.pdf]

Fig A – Blume et al.

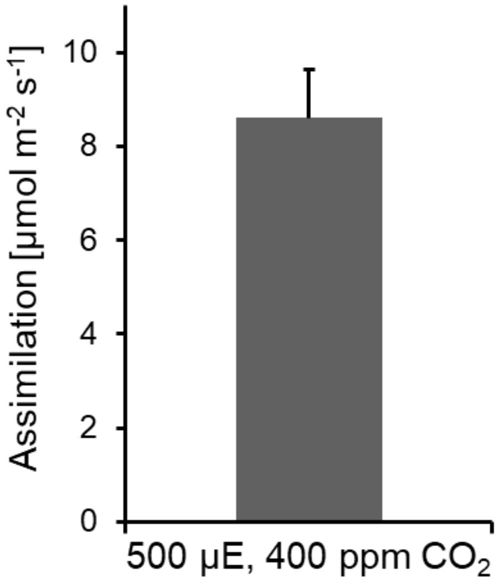

Fig A. CO_2 assimilation of 5-weeks-old *Arabidopsis* plants. Net CO_2 assimilation was recorded under ambient CO_2 conditions (400 ppm) and light saturating conditions (500 µE).

Fig B – Blume et al.

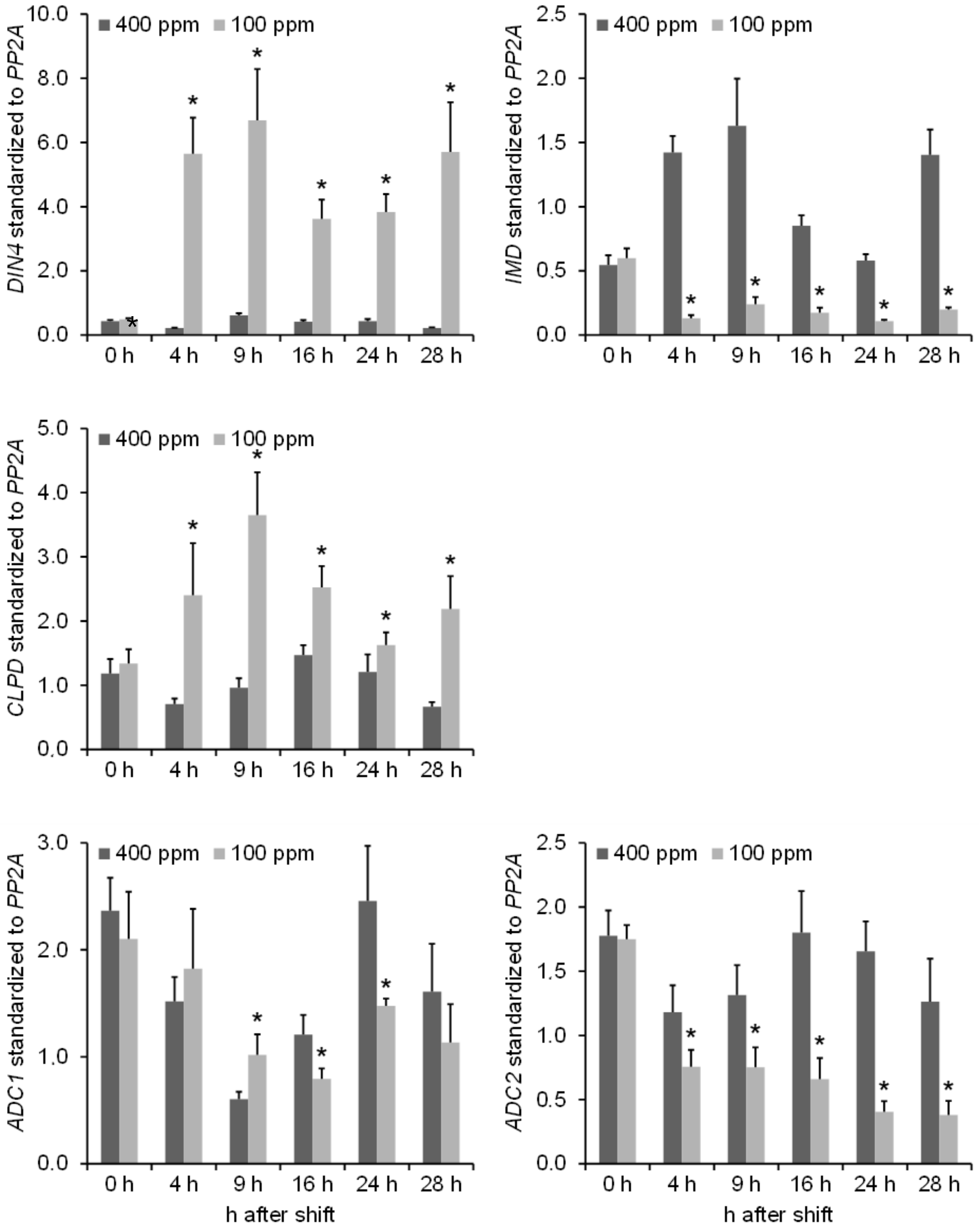

Fig B. Transcript levels of selected genes of leucine synthesis (*IMD*) and degradation (*DIN4*), protein degradation (*CLPD*), and polyamine synthesis (*ADC1, 2*) at 400 ppm and 100 ppm CO_2 standardized to *PP2A*. Data are the mean of five biological replicates ± SD. Significance was tested according to the two-tailed Student's t-test; values were changed to log_2 ratios to allow this test (* $p<0.05$) DIN4, Dark-induced 4; IMD, Isopropylmalate dehydrogenase; CLPD, caseinolytic protease D; ADC, arginine decarboxylase.

Fig C – Blume et al.

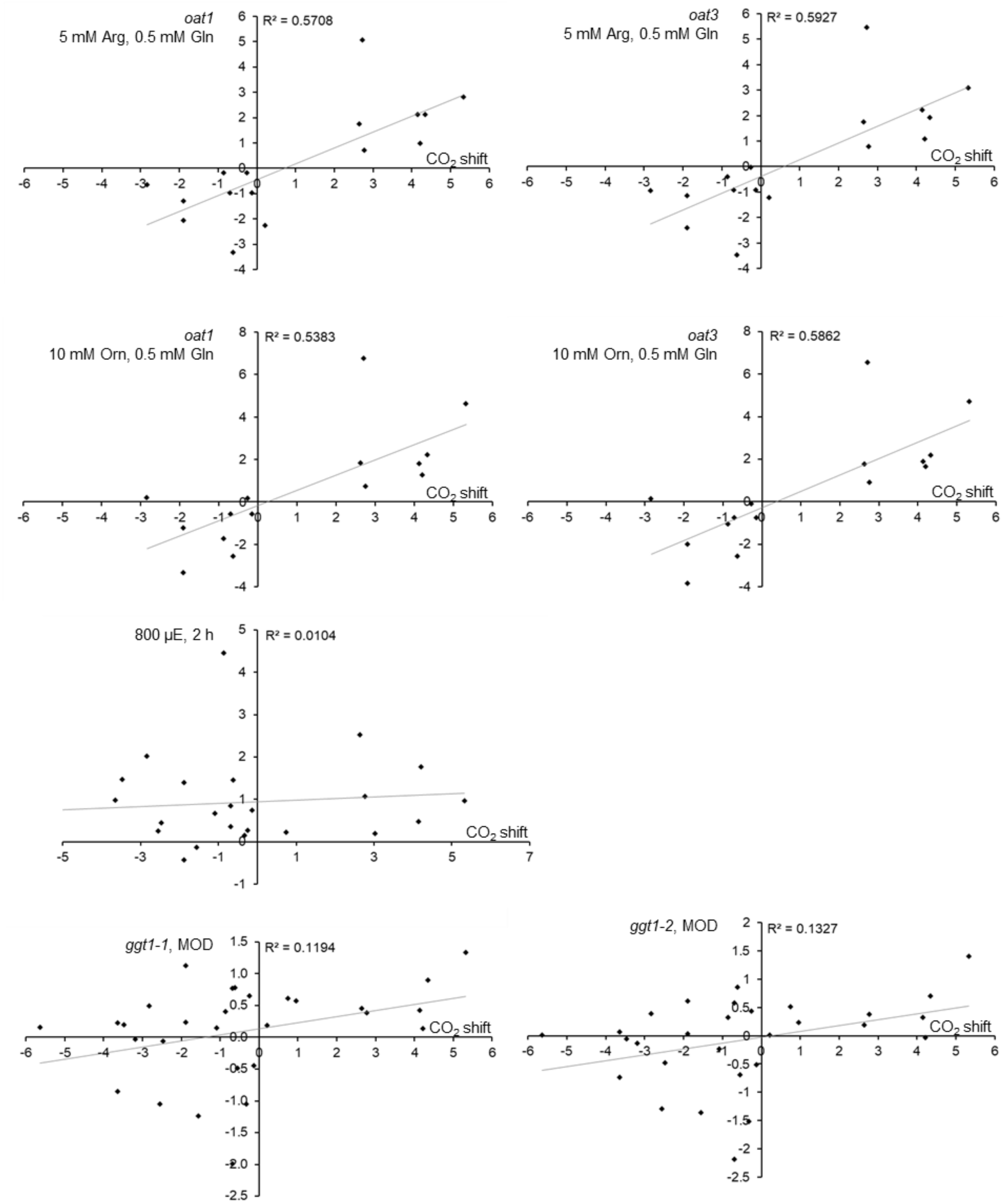

Fig C. Correlation of log2 metabolite changes between the low CO_2 shift and in response to different stresses/different *Arabidopsis* mutants. The fold changes are given in Fig 6.

Fig D – Blume et al.

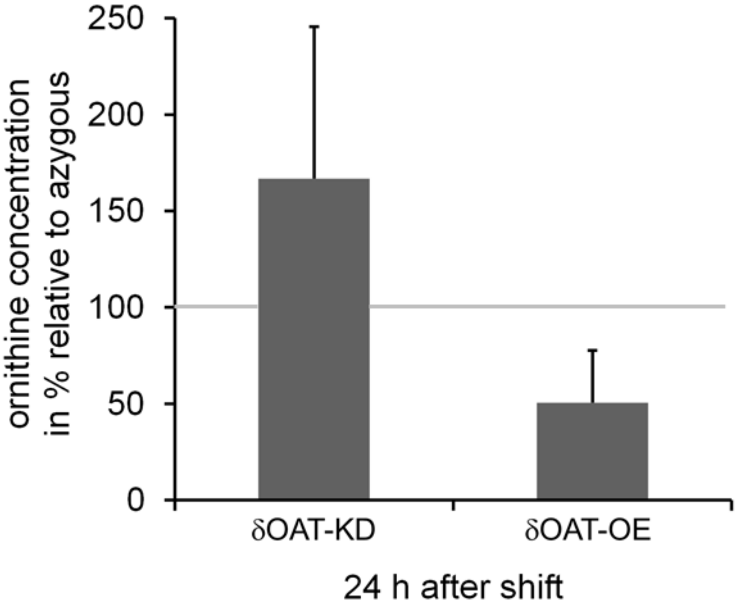

Fig D. Concentration of ornithine in knockdown (KD) and overexpression (OE) lines of *δOAT* after a shift from 400 ppm to 100 ppm CO_2 relative to azygous plants. Plant material was harvested 24 h after shifting the plants to low CO_2 concentrations. Data are the mean of three (KD) or two (OE) biological replicates ± SD. Grey line: wild type *δOAT* level.
